# High-speed running quadruped robot with a multi-joint spine adopting a 1DoF closed-loop linkage

**DOI:** 10.3389/frobt.2023.1148816

**Published:** 2023-03-24

**Authors:** Ojiro Matsumoto, Hiroaki Tanaka, Takumi Kawasetsu, Koh Hosoda

**Affiliations:** Adaptive Robotics Laboratory, Graduate School of Engineering Science, Osaka University, Toyonaka, Japan

**Keywords:** spine structure, closed-loop linkage, quadruped robot, high-speed running, bio-inspired, pelvic motion, quasi-quadruped robot

## Abstract

Improving the mobility of robots is an important goal for many real-world applications and implementing an animal-like spine structure in a quadruped robot is a promising approach to achieving high-speed running. This paper proposes a feline-like multi-joint spine adopting a one-degree-of-freedom closed-loop linkage for a quadruped robot to realize high-speed running. We theoretically prove that the proposed spine structure can realize 1.5 times the horizontal range of foot motion compared to a spine structure with a single joint. Experimental results demonstrate that a robot with the proposed spine structure achieves 1.4 times the horizontal range of motion and 1.9 times the speed of a robot with a single-joint spine structure.

## 1 Introduction

High-speed running is one of the important tasks that a quadruped robot should accomplish. Recent advances in actuators and computers have dramatically improved the mobility of quadruped robots. Quadruped robots such as Spot^®^ ^1^ and ANYmal^2^ can traverse stairs and uneven terrain by walking. Such robots can replace humans for tedious and potentially dangerous work such as terrain measurement, security, and cargo transport. However, current quadruped robots do not have the agility or high-speed running capabilities observed in quadruped animals. To expand the range of application of quadruped robots, many researchers have attempted to achieve high-speed running for such robots.

The implementing of a spine structure has attracted significant attention as a promising approach to increasing the speed of quadruped robots. Quadruped animals repeat flexion and extension of their spines in the sagittal plane during running (Hildebrand, 1959). In feline animals, the flexion and extension of the spine is pronounced during high-speed running and cheetahs can reach speeds of 29 m/s (104.4 km/h) by utilizing spine actuation (Sharp, 1997). Spine flexion and extension cause significant pelvic motion, including changes in the hip joint position (English, 1980) and tilt angle of the pelvis (Hildebrand, 1959; Schilling and Hackert, 2006) in the sagittal plane. The former expands the translational movement of the hindlimbs and the latter expands the rotational movement of the hindlimbs, thereby expanding the range of motion of the feet. Because expanding the range of motion of the feet generally increases running speed (Hildebrand, 1959; Schilling and Hackert, 2006), quadruped robots can achieve improved speed by implementing spine structures focused on changes in hip joint position and pelvic tilt angle caused by spine actuation.

A spine structure is generally composed of multiple joints and links to expand foot range of motion through the actuation of the spine structure. Several researchers have proposed single-joint spine structures that simplify spine actuation and can be mounted on a quadruped robot (Narioka et al., 2012; Khoramshahi et al., 2013; Zhao et al., 2013; Kawasaki et al., 2016; Chen et al., 2017; Chen et al., 2019; Fukuhara et al., 2020; Kim et al., 2021). Because these spine structures have only a single joint in the sagittal plane, they cannot achieve curved shapes similar to the spines of quadruped animals. Therefore, it is difficult to reproduce significant changes in hip joint position and pelvic tilt angle when utilizing a single-joint spine structure. Moreover, if a single-joint spine structure increases the actuation angle to achieve a wide horizontal foot range of motion, the clearance between the foot and the ground becomes shortened. The reduced clearance makes contact with the ground more likely and inhibits foot movement, resulting in a smaller foot range of motion. One study reported that even when a quadruped robot actuated a single-joint spine structure during running, the foot range of motion did not change significantly compared to when the robot ran without spine actuation and the robot was not able to achieve movement expansion of its limbs equivalent to that realized by quadruped animals (Khoramshahi et al., 2013).

A multi-joint spine structure must coordinate the rotation of each joint quickly and precisely during running. Underactuated cable driving is a common method for coordinating the joint rotation of a spine structure, where cables are placed across multiple joints and driven by a number of actuators smaller than the number of joints (Zhao et al., 2012; Seok et al., 2014; Eckert et al., 2015; Lei et al., 2022). Compared to the method of implementing actuators in each joint (Eckert et al., 2020; Li et al., 2020), this method can reduce the number of actuators to match that of a single-joint spine structure, thereby reducing the weight of robot. Additionally, the actuators that drive the cables can be located distally relative to the moving parts of the spine structure, thereby making the spine structure more compact. However, an underactuated system has redundant degrees of freedom (DoFs). During running, the spine structure must repeat extension and flexion in a short period and the spine structure receives large disturbance forces through the hindlimbs when the feet touch down. This makes it difficult to maintain the precision of the shape changes in the spine structure if redundant DoFs remain. Therefore, to reproduce changes in hip joint position and pelvic tilt angle quickly and precisely in each cycle of running, we must design a multi-joint spine structure that coordinates joint rotation without relying on a mechanism with redundant DoFs.

**FIGURE 1:**
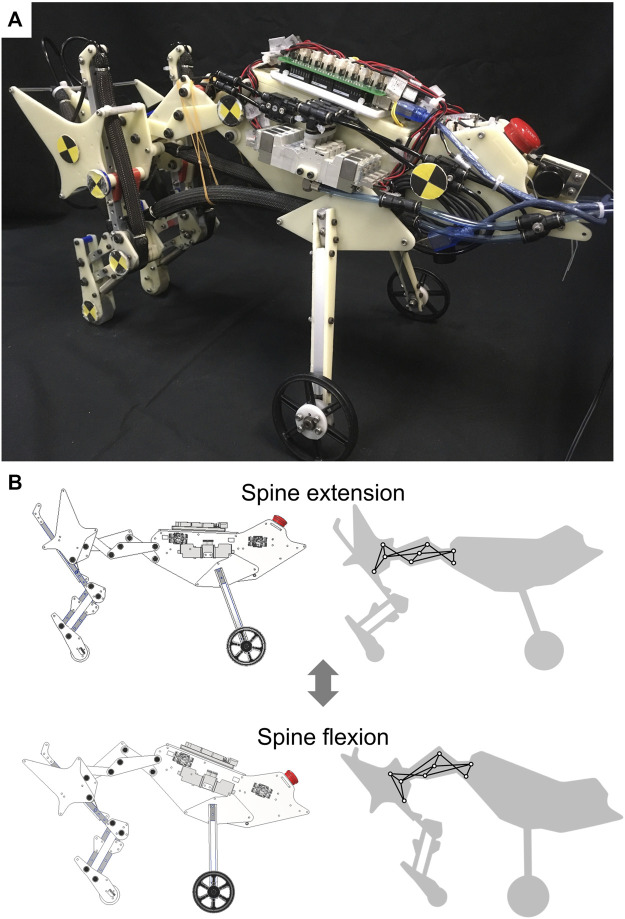
Quasi-quadruped robot with the proposed spine structure **(A)** Overview of the robot with the proposed spine structure. **(B)** Actuation of the proposed spine structure. The left images show a robot in a computer-aided design program and the right images show the adopted 1DoF linkage in the proposed spine structure.

In this paper, we propose a multi-joint spine structure that reproduces changes in hip joint position and pelvic tilt angle with a small number of DoFs and evaluate the running performance of a robot incorporating the proposed spine structure (Figure 1). The proposed spine structure consists of a 1DoF closed-loop linkage. This linkage provides a rotation chain for all joints and enables the spine structure to repeat extension and flexion quickly and precisely using only a few actuators. We embedded a point and link corresponding to the hip joint and pelvis in the linkage. The parameters of the linkage were defined to imitate the changes in hip joint position and pelvic tilt angle of a running feline animal. We defined the horizontal foot range of motion and running speed as the running performance metrics and compared performances between the proposed spine structure and a single-joint spine structure.

## 2 Feline-inspired spine design for a quadruped robot

First, we estimated changes in hip position and pelvic tilt angle in feline animals based on anatomical studies. Next, we selected a mechanism and corresponding parameters to reproduce the estimated hip joint position and pelvic tilt angle changes. The following subsections describe the estimation of changes in hip joint position and pelvic tilt angle in feline animals and the design of the proposed mechanism for a spine structure. Based on forward kinematics calculations, we also compare the horizontal foot range of motion achieved by the actuation of the proposed spine structure to that of a single-joint spine structure.

### 2.1 Pelvic motion in feline animals

**FIGURE 2:**
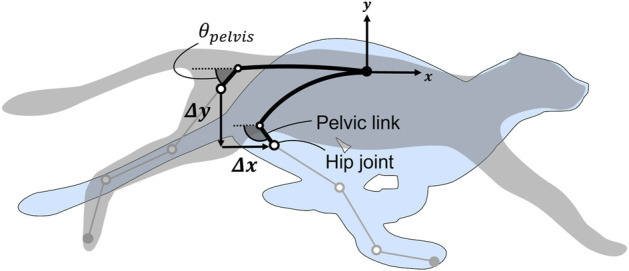
Change in the hip joint position and pelvic tilt angle during spine actuation. Δ*x* and Δ*y* denote the ranges of motion of the hip joint from full extension to full flexion of the spine. *θ*_*pelvis*_ denotes the angle of the pelvic link (i.e., pelvic tilt angle).

Figure 2 presents the changes in hip joint position and pelvic tilt angle observed during the spine actuation of a feline animal. Hereafter, the link corresponding to the pelvis will be referred to as the pelvic link. The propulsion direction is along the *x*-axis, and Δ*x* and Δ*y* denote the ranges of motion of the hip joint from full extension to full flexion of the spine. *θ*_*pelvis*_ denotes the angle of the pelvic link and corresponds to the pelvic tilt angle. Because spine flexion in quadruped animals mainly occurs posterior to the thoracic vertebrae (Schilling and Hackert, 2006), in this study, we limited the region of actuation of the spine structure to the area from the lumbar vertebrae to the pelvis. Additionally, because the hip joint is located on the pelvis, we estimated the position of the hip joint and changes in the pelvic tilt angle by reproducing the posture of the pelvis observed during feline running. We estimated the posture of the pelvis based on previous studies that investigated the length of the vertebrae and range of motion of the joints (English, 1980; Macpherson and Ye, 1998).

In a domestic cat with a body length of approximately 350 mm, the estimated values are |Δ*x*| = 75.1 mm and |Δ*y*| = 85.0 mm. The pelvic tilt angle is *θ*_*pelvis*_ = 41.3° during extension and *θ*_*pelvis*_ = 113.4° during flexion representing a change of 72.1°. The length of each vertebra and angles formed by each segment are listed in Supplementary Table S1 and Supplementary Table S2 of the Supplementary Material.

### 2.2 1DoF closed-loop linkage for coordinating joint rotation

**FIGURE 3:**
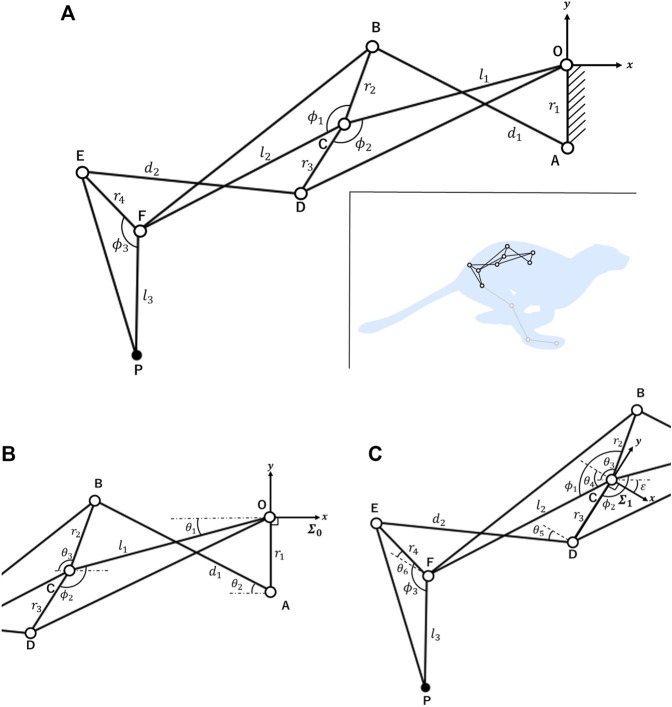
Kinematic model of the 1DoF closed-loop linkage **(A)** Overview of the closed-loop linkage. We implemented the spine structure by fixing the link OA to the center of the robot’s body. Each parameter takes a positive value. **(B)** Parameters *θ*_1_, *θ*_2_, and *θ*_3_ were added to calculate the coordinates of points A, B, C, and D in coordinate system *∑*_0_. Each *θ* is an angle relative to the *x*-negative axis and takes positive values clockwise and negative values counterclockwise to this axis. **(C)** Parameters *θ*_4_, *θ*_5_, and *θ*_6_ were added to calculate the coordinates of points E, F, and P in coordinate system *∑*_1_. The definition of each *θ* value is the same as described above.

Reproducing the changes in hip joint position and pelvic tilt angle estimated from a real feline animal requires the spine structure to have at least two or more joints. We adopted a 1DoF linkage (Murali et al., 2019) that is commonly applied in robot finger structures as a spine structure. This linkage provides a rotation chain for each joint and has several repeating units, each of which consists of a cross-four-bar linkage and triangular linkage (Matsumoto et al., 2019). Increasing the number of combined units provides many joints in the spine structure, enabling a wide range of hip joint position and pelvic tilt angle changes. In this study, we designed a linkage with three joints. Figure 3A presents the structure of the proposed spine. The origin O corresponds to the joint between the thoracic and lumbar vertebrae. Links OC and CF correspond to the lumbar vertebrae, link FP corresponds to the pelvic link, and point P corresponds to the hip joint. We can calculate the position of point P and the angle of link FP by using the forward kinematics approach described below.

In Figures 3B, C, only *θ*_1_ is a variable and each *θ* is a function of *θ*_1_. Note that each *θ* is an angle corresponding to the *x*-negative axis that takes positive values clockwise and negative values counterclockwise with respect to this axis. First, we calculated the coordinates of points A, C, D, and B from the coordinate system *∑*_0_ in Figure 3B. Points A, C, and D are relatively easy to calculate, whereas point B must be computed through equations based on the constraints of the four-bar linkage OABC. Part of the calculation of the coordinates of each point in a four-bar linkage is based on previous research (Norton, 2008). Equations based on the constraints of the four-bar linkage OABC in the coordinate system *∑*_0_ can be defined as follows:

$$l_{1}\text{cos}\left(\pi +\theta_{1}\right)-d_{1}\text{cos}\left(\pi +\theta_{2}\right)+r_{2}\text{cos}\left(\pi +\theta_{3}\right)=0,$$ (1)

$$l_{1}\text{sin}\left(\pi +\theta_{1}\right)-d_{1}\text{sin}\left(\pi +\theta_{2}\right)+r_{2}\text{sin}\left(\pi +\theta_{3}\right)+r_{1}=0.$$ (2)

By combining Eqs 1, 2 to remove *θ*_3_, the constraint of the four-bar linkage OABC can be described as follows:

$$r_{1}^{2}+l_{1}^{2}+d_{1}^{2}-r_{2}^{2}-2r_{1}l_{1}\text{sin}\theta_{1}+2d_{1}\left(r_{1}-l_{1}\text{sin}\theta_{1}\right)\text{sin}\theta_{2}-2l_{1}d_{1}\text{cos}\theta_{1}\text{cos}\theta_{2}=0.$$ (3)

To calculate *θ*_2_, we define *α*_0_, *β*_0_, and *γ*_0_ as follows:

$$\alpha_{0}=r_{1}^{2}+l_{1}^{2}+d_{1}^{2}-r_{2}^{2}-2r_{1}l_{1}\text{sin}\theta_{1}+2l_{1}d_{1}\text{cos}\theta_{1},$$ (4)

$$\beta_{0}=2d_{1}\left(r_{1}-l_{1}\text{sin}\theta_{1}\right),$$ (5)

$$\gamma_{0}=r_{1}^{2}+l_{1}^{2}+d_{1}^{2}-r_{2}^{2}-2r_{1}l_{1}\text{sin}\theta_{1}-2l_{1}d_{1}\text{cos}\theta_{1}.$$ (6)

Based on Eqs 4–6, Eq. 3 can be rewritten as

$$\text{tan}\frac{\theta_{2}}{2}=\frac{-\beta_{0}\pm \sqrt{\beta_{0}^{2}-\alpha_{0}\gamma_{0}}}{\alpha_{0}}.$$ (7)

According to Eq. 7, *θ*_2_ has two solutions. One solution corresponds to the cross-four-bar linkage OABC and the other corresponds to the open-four-bar linkage OABC. It is possible to prove that the part corresponding to the “±” in Eq. 7 takes “+” when the shape of the four-bar linkage OABC opens and “−” when it crosses according to previous research (Chase and Mirth, 1993). The corresponding details are provided in section 2 of the Supplementary Material. Because the four-bar linkage OABC in the proposed spine structure always crosses, *θ*_2_ can be determined as follows:

$$\theta_{2}=2{\text{tan}}^{-1}\frac{-\beta_{0}-\sqrt{\beta_{0}^{2}-\alpha_{0}\gamma_{0}}}{\alpha_{0}}.$$ (8)

By combining Eqs 1, 2, 8, *θ*_3_ can be calculated as follows:

$$\theta_{3}={\text{tan}}^{-1}\frac{l_{1}\text{sin}\theta_{1}-d_{1}\text{sin}\theta_{2}-r_{1}}{l_{1}\text{cos}\theta_{1}-d_{1}\text{cos}\theta_{2}}.$$ (9)

Therefore, we can calculate the coordinates of point B in coordinate system *∑*_0_ by using Eq. 9.

The same procedure can be applied to calculate the coordinates of points E, F, and P in Figure 3C, as viewed from coordinate system *∑*_1_. The origin of coordinate system *∑*_1_ is point C and the *y*-axis is the same as that of the link CD. *θ*_4_, *θ*_5_, and *θ*_6_ in coordinate system *∑*_1_ geometrically correspond to *θ*_1_, *θ*_2_, and *θ*_3_ in coordinate system *∑*_0_. The rotation angle of coordinate system *∑*_1_ from coordinate system *∑*_0_ is described as follows:

$$\epsilon =-\phi_{2}+\theta_{1}+\frac{\pi }{2}.$$ (10)

*θ*_4_ is expressed as follows:

$$\theta_{4}=\phi_{1}+\phi_{2}-\theta_{1}+\theta_{3}-\frac{\pi }{2}.$$ (11)

Equations based on the constraints of the four-bar linkage CDEF in coordinate system *∑*_1_ can be written as follows:

$$l_{2}\text{cos}\left(\pi +\theta_{4}\right)-d_{2}\text{cos}\left(\pi +\theta_{5}\right)+r_{4}\text{cos}\left(\pi +\theta_{6}\right)=0,$$ (12)

$$l_{2}\text{sin}\left(\pi +\theta_{4}\right)-d_{2}\text{sin}\left(\pi +\theta_{5}\right)+r_{4}\text{sin}\left(\pi +\theta_{6}\right)+r_{3}=0.$$ (13)

To calculate *θ*_5_, we define *α*_1_, *β*_1_, and *γ*_1_ as follows:

$$\alpha_{1}=r_{3}^{2}+l_{2}^{2}+d_{2}^{2}-r_{4}^{2}-2r_{3}l_{2}\text{sin}\theta_{4}+2l_{2}d_{2}\text{cos}\theta_{4},$$ (14)

$$\beta_{1}=2d_{2}\left(r_{3}-l_{2}\text{sin}\theta_{4}\right),$$ (15)

$$\gamma_{1}=r_{3}^{2}+l_{2}^{2}+d_{2}^{2}-r_{4}^{2}-2r_{3}l_{2}\text{sin}\theta_{4}-2l_{2}d_{2}\text{cos}\theta_{4}.$$ (16)

Because the four-bar linkage CDEF in the proposed spine structure always crosses, *θ*_5_ can be determined by using Eqs. 14–16 as follows:

$$\theta_{5}=2{\text{tan}}^{-1}\frac{-\beta_{1}-\sqrt{\beta_{1}^{2}-\alpha_{1}\gamma_{1}}}{\alpha_{1}}.$$ (17)

By combining Eqs. 11–13, 17, *θ*_6_ can be calculated as follows:

$$\theta_{6}={\text{tan}}^{-1}\frac{l_{2}\text{sin}\theta_{4}-d_{2}\text{sin}\theta_{5}-r_{3}}{l_{2}\text{cos}\theta_{4}-d_{2}\text{cos}\theta_{5}}.$$ (18)

Through the calculations above, we can calculate the coordinates of points E, F, and P in coordinate system *∑*_1_ by using Eq. 18. Finally, we can calculate the coordinates of all points in coordinate system *∑*_0_ by transferring the coordinates of points E, F, and P in coordinate system *∑*_1_ into coordinate system *∑*_0_ using a homogeneous transformation matrix based on Eq. 10.

By adjusting the length of each link, joint angles, and domain of the variable *θ*_1_, we derived a set of parameters for the linkage that reproduced the changes in the hip joint position and pelvic tilt angle estimated in the previous section. These parameters are *l*_1_ = 70.0 mm, *l*_2_ = 70.0 mm, *l*_3_ = 37.5 mm, *r*_1_ = 25.0 mm, *r*_2_ = 25.0 mm, *r*_3_ = 25.0 mm, *r*_4_ = 25.0 mm, *d*_1_ = 66.4 mm, *d*_2_ = 66.4 mm, *ϕ*_1_ = 137.5°, *ϕ*_2_ = 137.5°, and *ϕ*_3_ = 135.0°. Based on the |Δ*x*|, |Δ*y*|, and *θ*_*pelvis*_ defined in Figure 2, this linkage shifts the hip joint by |Δ*x*| = 75.0 mm and |Δ*y*| = 86.4 mm when *θ*_1_ changes from 0.0° to 25.0° during the extension-to-flexion transition of the spine. The pelvic tilt angle is *θ*_*pelvis*_ = 45.0° during extension and *θ*_*pelvis*_ = 122.0° during flexion, representing a change of 77.0°.

### 2.3 Evaluation of foot range of motion

**FIGURE 4:**
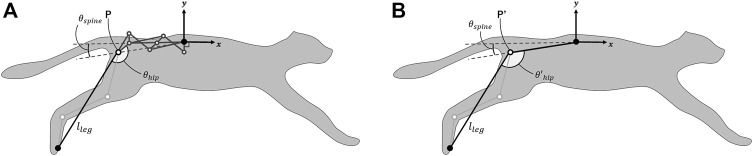
Parameters of each spine structure for comparing the horizontal foot range of motion achieved by spine actuation **(A)** Parameters of the proposed spine structure. Point P indicates the hip joint. The length of the leg approximated by a single link is *l*_*leg*_ and the angle formed by the leg link and pelvic link is *θ*_*hip*_. *θ*_*spine*_ is the angle between the *x*-negative axis and the line connecting the origin to the hip joint, which indicates spine actuation. **(B)** Parameters in the single-joint spine structure. Point P′ indicates the hip joint. The definitions of *l*_*leg*_ and *θ*_*spine*_ are the same as described above. $\theta_{\mathrm{hip}}^{\prime }$ is the angle between the leg link and spine link.

We compared the horizontal foot range of motion caused by spine actuation between the proposed spine structure and a single-joint spine structure. Figure 4 presents the parameters for each spine structure. The single-joint spine structure consists of a single link connecting the origin at the center of the body to P′, which represents the hip joint. The length of this link is the distance between the origin and P in the proposed spine during extension. The length of the leg approximated by a single link is *l*_*leg*_ and the angles formed by the leg link and spine link are *θ*_*hip*_ and $\theta_{\mathrm{hip}}^{\prime }$ , respectively. To focus on the foot range of motion depending on spine actuation, we set *l*_*leg*_, *θ*_*hip*_, and $\theta_{\mathrm{hip}}^{\prime }$ to fixed values in this comparison. We set *l*_*leg*_ = 200.0 mm, *θ*_*hip*_ = 155.7°, and $\theta_{\mathrm{hip}}^{\prime }=120.0{}^{\circ}$ so that the position of the foot in both spine structures is the same when the spine is fully extended. For each spine structure, we defined *θ*_*spine*_ as the angle formed by the *x*-negative axis and a line connecting the origin to the hip joint to represent spine actuation. The domain of *θ*_*spine*_ is 9.0°–50.9°, which corresponds to the shape change when the *θ*_1_ described in the previous section changes from 0.0° to 25.0°.

**FIGURE 5:**
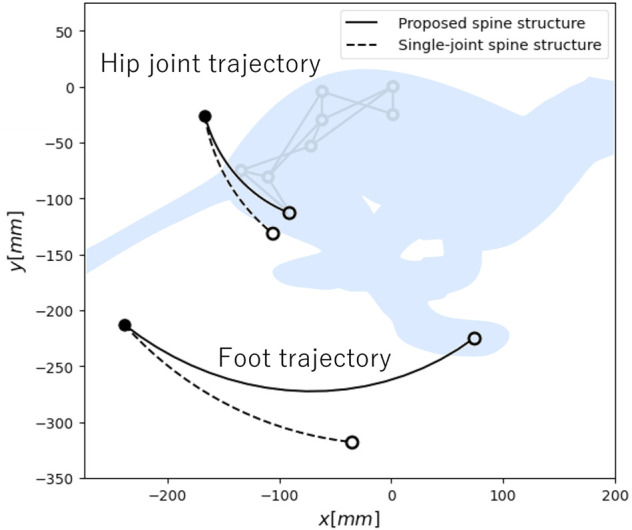
Hip joint and foot trajectory during spine actuation in each spine structure. Here, • and ◦ denote the coordinates when the spine structure fully extends and fully flexes, respectively.

Figure 5 presents the hip joint and foot trajectory for each spine structure when *θ*_*spine*_ changes. The *x*-axis represents the direction of propulsion. When *l*_*leg*_, *θ*_*hip*_, and $\theta_{\mathrm{hip}}^{\prime }$ are fixed values, the horizontal range of motion of the hip joint and foot of the proposed spine structure are 75.0 mm and 312.3 mm, respectively, and those of the single-joint spine structure are 60.3 mm and 203.4 mm, respectively. Therefore, the proposed spine structure can increase the foot range of motion in the propulsive direction by a factor of 1.5 compared to the single-joint spine structure. We determined that 86.5% of the difference in the foot range of motion between the proposed spine structure and single-joint spine structure can be attributed to the angular change of the pelvic link. Furthermore, the proposed spine structure shows a smaller vertical displacement of the foot. This means the proposed spine structure can maintain a greater clearance between the foot and the ground during the spine actuation. Based on this result, it is important to consider not only the horizontal range of motion of the hip joint, but also the angular change of the pelvic link when designing a spine structure to expand the foot range of motion in the propulsive direction.

## 3 Quasi-quadruped robot

To confirm whether the proposed spine structure can increase running speed by expanding the foot range of motion, we developed the robot presented in Figure 1, which can implement both the proposed spine structure and a single-joint spine structure. To focus on the propulsion function of the hindlimbs and spine structure, we assumed that the forelimb function is to maintain the body height and implemented front tires instead of the forelimbs. We employed McKibben-type pneumatic artificial muscles (PAMs), which have low weight and a large output force, as actuators for the robot. The following sections describe the hardware design of the quasi-quadruped robot, arrangement of PAMs, and actuation pattern of PAMs.

### 3.1 Hardware design

The specifications of the robot are provided in Table 1. We designed each link length and joint range of motion based on the body mechanics of a domestic cat (English, 1980; Ekeberg and Pearson, 2005). The robot consists mainly of POM (engineering plastic) plates and square aluminum pipes. The robot uses a microcontroller (Arduino Mega) and solenoid valves (VQZ1321-6L1-C6, SMC Co.) to control the PAMs, and compressed air and a power source are supplied to the robot from external sources. A pantograph structure (Witte et al., 2000) is applies to the hindlimbs, which can reproduce animal-like hindlimb motion and reduce the number of DoFs and actuators in the limbs. This mechanism allows the knee and ankle joints to be coordinated.

**TABLE 1:** Key characteristics of the robot.

Property	Value
Length × Height × Width	540 × 375 × 235 mm
Total weight	3.9 kg
Number of valves	8
Number of muscles	8
Femur length	90 mm
Tibia length	100 mm
Foot length	60 mm

**FIGURE 6:**
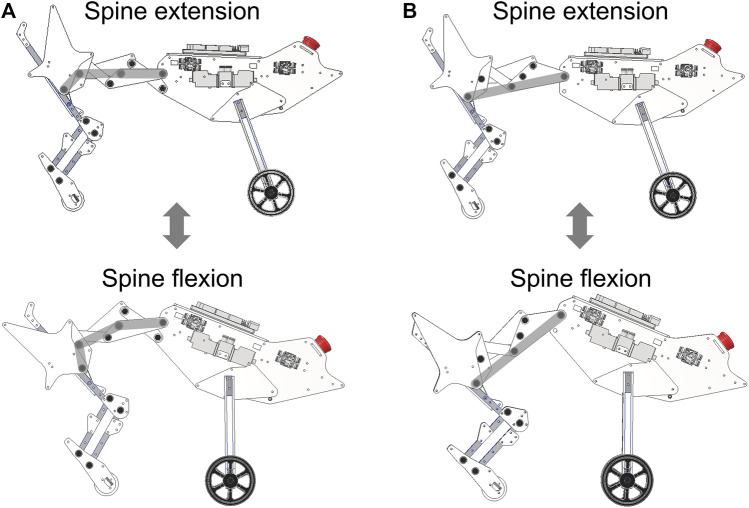
Robot equipped with two types of spine structures, namely, the proposed spine structure and a single-joint spine structure. The gray lines indicate the shape of the spine structure during extension and flexion. **(A)** Robot equipped with the proposed spine structure. **(B)** Robot equipped with the single-joint spine structure.

The robot can implement a single-joint spine structure by locking the shape change of the linkage in the proposed spine structure and allowing the joints at the base of the linkage to move freely. The robot configured with the proposed spine structure and with a single-jointed spine structure is presented in Figure 6. This spine structure switching allowed us to conduct running experiments using a single robot.

### 3.2 Arrangement of the muscles

PAMs are actuators that can contract and expand similar to biological muscles. When injecting compressed air into a PAM, it inflates and generates a large amount of tension. Compared to a pneumatic cylinder, a PAM is lighter and more robust, even when a radial deformation force is applied. These features make PAMs suitable actuators for robots that must move dynamically. The contraction and extension of the PAMs are controlled by solenoid valves that connect the PAMs to the air tank. When a solenoid valve supplies air to a PAM, it causes the PAM to contract, and when the valve exhausts air, it causes the PAM to extend.

**FIGURE 7:**
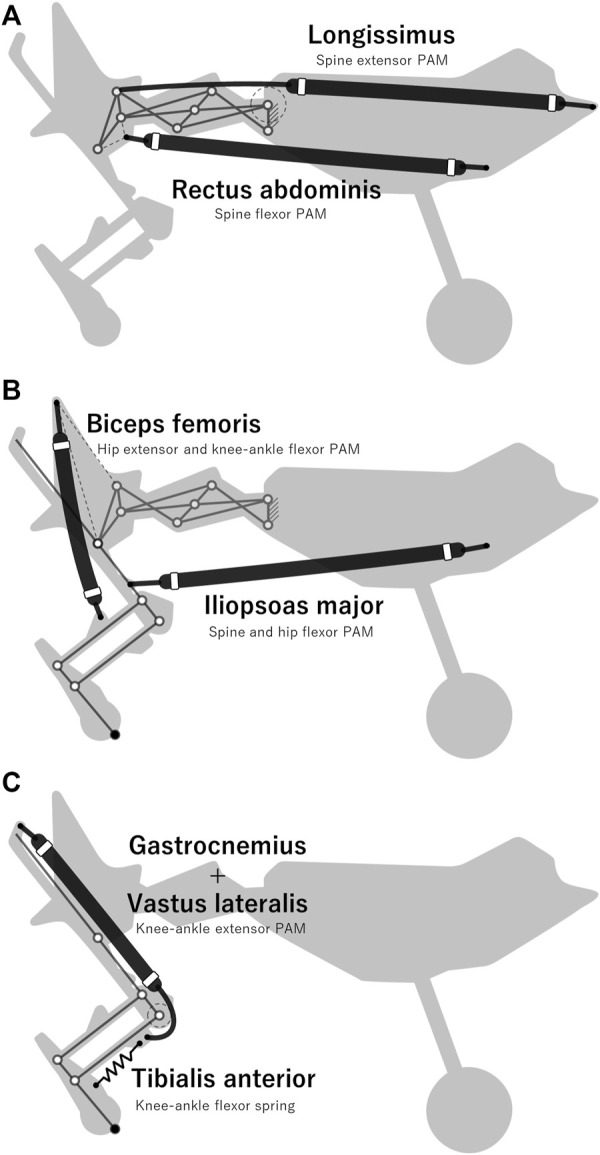
Arrangement of PAMs implemented in the designed robot **(A)** Muscles that actuate the spine structure. **(B)** Muscles that mainly actuate the hip joint. **(C)** Muscles that actuate the knee-ankle joint.

We fabricated all the PAMs implemented in our robot with a polyester shell, natural rubber tubing, adaptors, and metal wire. Based on previous studies on anatomy and robotics (Macpherson and Ye, 1998; Galbusera and Bassani, 2019; Masuda and Ishikawa, 2020), we selected muscles that are considered to be particularly important among the many muscles in the body and implemented them in our robot. The selected muscles and their arrangement are presented in Figure 7. The longissimus and rectus abdominal muscles shown in Figure 7A actuate the spine structure, changing *θ*_spine_ between 9.0° and 50.9°.

### 3.3 Actuation pattern of PAMs

**FIGURE 8:**
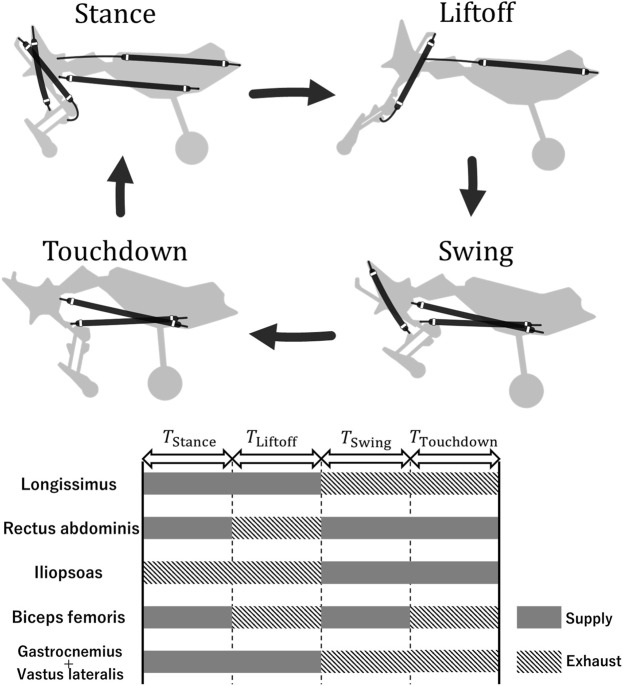
Actuation pattern of the PAMs. The top figure presents only the PAMs contracting in each phase.

In the robot running experiments, the robot actuated each PAM according to a feedforward rule to realize running. Based on previous studies (Ekeberg and Pearson, 2005), we classified the phases of each PAM actuation during one cycle into four phases: stance, liftoff, swing, and touchdown. The actuation pattern of each PAM in each phase was determined by referring to the electromyography pattern of a domestic cat during running, as shown in Figure 8 (English, 1980; Kandel et al., 2000). If the time of one cycle is *T* ms, then *T* = *T*_Stance_ + *T*_Liftoff_ + *T*_Swing_ + *T*_Touchdown_.

In the running experiments described below, *T* was fixed to compare the speeds achieved by the robot for each spine structure. We set the period *T* = 330 ms so that the stride frequency was approximately 3 Hz because previous studies have noted that the stride frequency of a running cheetah at its maximum speed is approximately 3 Hz (Hudson et al., 2012). The duration of each phase was set *T*_Stance_ = 115 ms, *T*_Liftoff_ = 82 ms, *T*_Swing_ = 115 ms, and *T*_Touchdown_ = 16 ms. Each phase corresponds to 35%, 25%, 35%, and 5% of the period *T*, respectively (rounded down to the nearest whole number). This combination of durations is one of the fastest combinations achieved for the robot with each spine structure in the experimental environment described below. Further details regarding the selection of durations are provided in section 3 of the Supplementary Material.

## 4 Setup for running experiments

**FIGURE 9:**
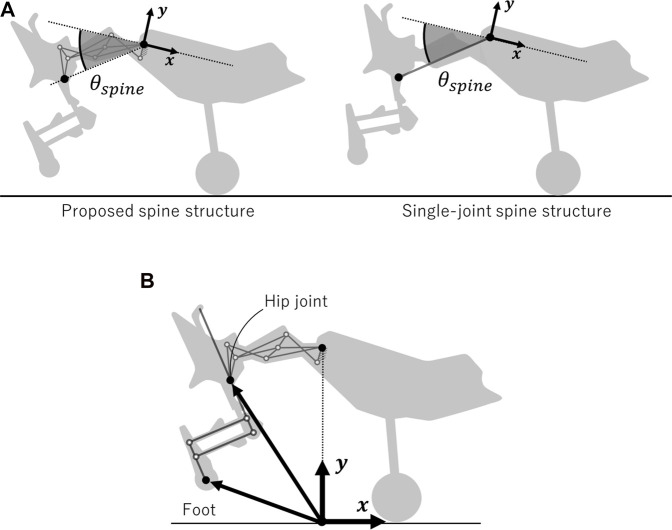
Parameter settings for comparing the running performance of the robot with each spine structure **(A)** Definition of *θ*_*spine*_, which represents the range of motion of the spine in the proposed and single-joint spine structures. *θ*_*spine*_ is the angle between the *x*-negative axis and the line connecting the origin to the hip joint. **(B)** Arrows indicate the hip joint and foot position as viewed from the origin.

We confirmed whether differences in the horizontal foot range of motion depending on the spine structure led to differences in the running speed of the robot. There were two points to be confirmed in our experiments. The first was to ensure that the robot equipped with each spine structure ran with full spine actuation. As shown in Figure 9A, we defined *θ*_*spine*_ for each spine structure. As mentioned previously, each spine structure was designed to allow *θ*_*spine*_ to change from 9.0° to 50.9°, which corresponds to the extension-to-flexion transition of the spine. By checking the range of *θ*_*spine*_, we confirmed that the proposed spine structure could change the position of the hip joint and angle of the pelvic link as designed. The second point was to confirm whether the robot equipped with the proposed spine structure expanded the horizontal foot range of motion during running compared to the robot equipped with a single-joint spine structure. We defined the coordinates of the origin of the robot as shown in Figure 9B. During the stance period, we compared the horizontal range of motion of the hip joint and foot with respect to the origin between the robot with the proposed spine structure and single-joint spine structure.

Robot running experiments were conducted on a treadmill whose speed could be changed in increments of every 0.1 km/h. The robot moved only in the sagittal plane based on restriction by transparent plates on both sides of the robot. The running speed of the robot was defined as the treadmill speed when the robot moved on the treadmill for more than 10 s. The robot was supplied with compressed air (0.65 MPa) and an external power source for its electronic components. The air tube and power cable were adjusted such that their tension did not affect the movement of the robot.

Based on the definition of running speed described above, we measured the robot’s movement during its 10 s run at a constant speed for five trials for each spine structure. A camera (RX100VII, Sony Co.) was used to capture the movement of the robot in the sagittal plane through a transparent plate at 120 fps. We analyzed the data using Kinovea (Charmant and contributors, 2021). To calculate the means and standard deviations of the data, we used data from five consecutive cycles of each trial (i.e., 25 cycles).

## 5 Results

**FIGURE 10:**
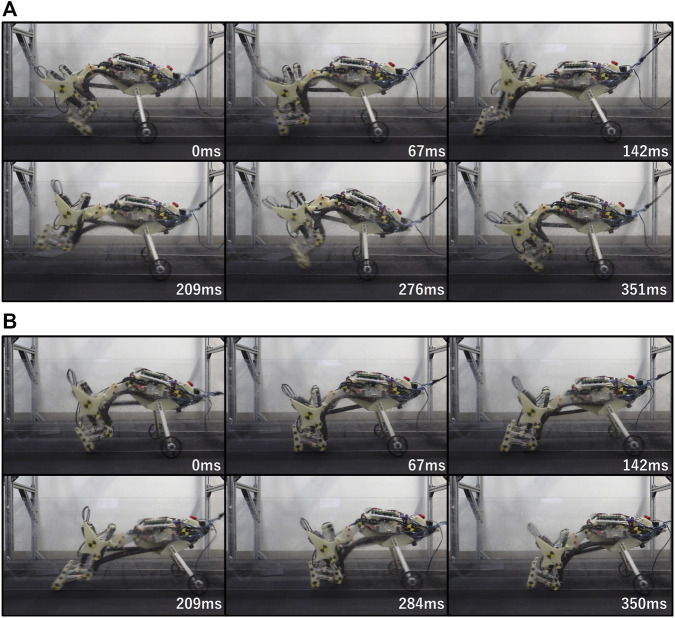
Snapshots of the running robot with each spine structure **(A)** Snapshots of the running robot with the proposed spine structure. The running speed is 1.61 m/s (5.8 km/h) **(B)** Snapshots of the running robot with the single-joint spine structure. The running speed is 0.83 m/s (3.0 km/h).

Figure 10 presents snapshots of the running robot for each spine structure. Supplementary Video S1 presents the operation of the robot equipped with the proposed spine structure and Supplementary Video S2 presents the operation of the robot equipped with a single-joint spine structure. The measurement results reveal that the robot with the proposed spine structure achieves a running speed of 1.61 m/s (5.8 km/h), and the robot with the single-joint spine structure achieves a running speed of 0.83 m/s (3.0 km/h). By multiplying each speed by the period of 330 ms, the stride lengths were determined to be 53.1 mm and 27.5 mm, respectively. The robot with the proposed spine structure did not cause its knees to collide with the ground during the stance period, whereas the robot with a single-joint spine structure often experienced knee-ground collision. Knee-ground collision occurred at 350 ms, as shown in Figure 10B. The mean stance duration of the robot with the proposed spine structure was 142 ms with a standard deviation of 13 ms, whereas the mean stance duration of the robot with the single-joint spine structure was 173 ms with a standard deviation of 12 ms.

**FIGURE 11:**
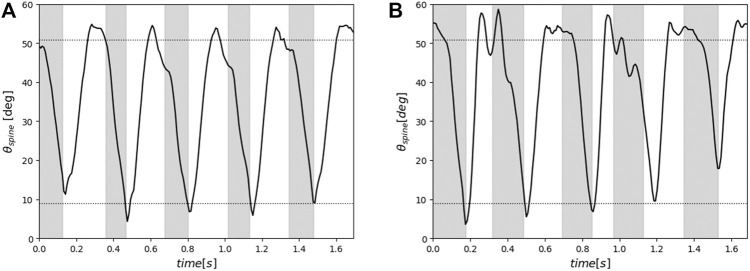
Range of motion of each spine structure actuation during five running cycles. The gray and white sections represent the stance and swing periods, respectively, and the dashed lines indicate the minimum (9.0 deg) and maximum (50.9 deg) values of *θ*_*spine*_ **(A)** Range of motion of the proposed spine structure. **(B)** range of motion of the single-joint spine structure.

Figure 11 presents the range of motion of each spine structure actuation during five cycles of robot running. The horizontal axis represents time and the vertical axis represents the change in *θ*_*spine*_, which indicates the actuation of the spine. The gray and white sections represent the stance and swing periods, respectively, and the dashed lines indicate the minimum (9.0°) and maximum (50.9°) values of *θ*_*spine*_. In Figures 11A, B, one can see that the robot equipped with each spine structure operates by actuating its spine to reach the maximum and minimum *θ*_*spine*_ in most cases. This result confirms that the robot with the proposed spine structure achieves the designed change in hip joint position and pelvic link tilt angle during running.

**FIGURE 12:**
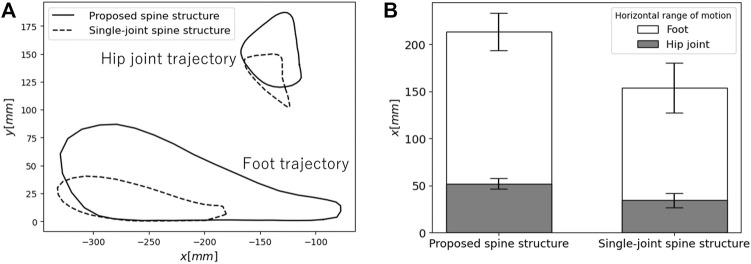
Comparison of the ranges of motion of the hip joint and foot between each spine structure **(A)** Average trajectories of the hip joint and foot as viewed from the robot origin during running. **(B)** Horizontal range of motion of the hip joint and foot during the stance phase.

Figure 12A presents the average trajectories of the hip joint and foot relative to the robot’s origin during running. One can see that the proposed spine structure significantly increases the foot range of motion during both the stance and swing phases, particularly in the propulsive direction. Figure 12B presents the ranges of motion of the hip joint and foot during the stance period. In the proposed spine structure, the mean range of motion of the hip joint is 52.1 mm with a standard deviation of 5.6 mm and that of the foot is 213.2 mm with a standard deviation of 19.8 mm. In the single-joint spine structure, the mean range of motion of the hip joint is 34.3 mm with a standard deviation of 7.4 mm and that of the foot is 154.0 mm with a standard deviation of 26.3 mm.

## 6 Discussion

In this paper, we proposed a spine structure that expands the horizontal foot range of motion by imitating feline pelvic motion to achieve high-speed running for a quadruped robot. Forward kinematics calculations revealed that the proposed spine structure could theoretically provide a 1.5 times greater horizontal foot range of motion compared to a single-joint spine structure. In experiments on a robot equipped with each spine structure, the robot equipped with the proposed spine structure achieved a 1.4 times greater horizontal foot range of motion and 1.9 times greater speed than the robot equipped with a single-joint spine structure. We consider that there are three reasons why the robot equipped with the proposed spine structure ran faster than the robot equipped with a single-joint spine structure. First, the proposed spine structure can expand the foot range of motion by utilizing changes in hip position and pelvic tilt angle, as designed. Second, the stance duration of the robot equipped with the proposed spine structure is shorter than that of the robot equipped with a single-joint spine structure, despite a wider foot range of motion. The robot with the proposed spine structure may have exerted more force on the ground through its foot. If the robot increases the impulse exerted on the ground per unit of time, the stance duration becomes shorter (Park and Kim, 2015). The robot equipped with the proposed spine structure can not only move its foot a greater horizontal distance, but can also achieve foot movement in a shorter timeframe during the stance period, resulting in a running speed 1.9 times greater than that of the robot with a single-joint spine structure. Third, the robot with a single-joint spine structure may have reduced running speed based on collisions between its knees and the ground. The disruption of the robot’s running due to the knee-ground collision can be seen in the waveforms perturbations around 0.3 s and 1.0 s in Figure 11B. It seems that the small clearance between the ground and the foot and the lack of the horizontal foot range of motion induce knee-ground collisions during running. The large clearance between the foot and the ground and the wide horizontal foot range of motion shown in Figure 5 would allow a robot with the proposed spine structure to prevent knee-ground collision during running.

The proposed spine structure can be used as a template for designing spine structures for high-speed running. Few studies have focused on the design theory of the spine structures required for high-speed running. Eckert et al. compared the performance of a robot equipped with a multi-joint spine structure to that of a robot equipped with a single-joint spine structure (Eckert et al., 2015). They found that the robot with a multi-joint spine structure ran slower than the robot with a single-joint spine structure. They noted that there are design theory challenges in both the limb and spine structure to utilize a multi-joint spine structure. Our robot used a pantograph structure with limbs similar to those of theirs and our robot equipped with a multi-joint spine structure ran faster than a robot with a single-joint spine structure. This indicates that the proposed spine structure can improve the running speed of a robot independently of its limb structure. Lei et al. demonstrated that the greater the number of joints in the spine structure, the faster the movement speed of a robot with a spine structure (Lei et al., 2022). They increased the number of joints from one to five, which increased the robot speed by a factor of 1.5. Our spine structure can increase the speed of robot by a factor of 1.9 while only increasing the number of joints from one to three. We believe that a method that reproduces pelvic motion can increase the foot range of motion more effectively than a method that increases the number of spine joints, thereby increasing the overall speed of the robot. Furthermore, the proposed spine structure is actuated by pneumatic artificial muscles, which can provide elasticity to the spine structure. Quadruped animals have elasticity in their spine (Alexander et al., 1985). Previous studies have suggested that the elasticity of the spine help to absorb disturbances and store and release energy (Kani et al., 2011; Tsujita and Miki, 2011; Pusey et al., 2013; Takuma et al., 2017; Duperret and Koditschek, 2017). Therefore, understanding how quadrupeds utilize the elasticity of the spine by using the robot developed in this study will further improve the locomotion performance of quadruped robots.

There are two ways to improve the running speed of a robot using the proposed spine structure further. The first is to increase the horizontal foot range of motion further by adjusting the parameters of the linkage. Here, we determined the parameters of the linkage for the proposed spine structure to imitate the pelvic motion of domestic cats. By searching for parameters of the linkage to maximize the horizontal foot range of motion, the robot should be able to achieve a longer foot range of motion during high-speed running. The second method is to reduce the stance duration by optimizing the forces generated at the foot based on the actuation of the spine structure. Reducing the stance duration means that the robot must support its body for a shorter time and generate greater force at the foot instantaneously (Park and Kim, 2015). Because previous studies have pointed out that actuating a spine structure increases the ground reaction force of a robot (Kawasaki et al., 2016), we can optimize the force generated at the foot by changing the parameters of the linkage. Even if the stance time is reduced, the foot range of motion during the stance should not be reduced. Therefore, linkage parameters must be explored to maximize both the force generated at the foot and the foot range of motion.

## 7 Conclusion

In this paper, we proposed a spine structure that expands the horizontal foot range of motion of a quadruped robot by imitating feline pelvic motion to achieve high-speed running motion. The proposed spine structure uses multiple joints to realize pelvic motion and coordinates the rotation of each joint using a 1DoF closed-loop linkage. Forward kinematics calculations revealed that the proposed spine structure can theoretically achieve a 1.5 times greater horizontal foot range of motion compared to a single-joint spine structure. In experiments on a robot equipped with each spine structure, the robot equipped with the proposed spine structure achieved a 1.4 times greater horizontal foot range of motion and 1.9 times greater speed than the robot equipped with a single-joint spine structure. The experimental results demonstrated that the robot equipped with the proposed spine structure increases foot range of motion and improves speed by imitating the pelvic motion of a feline and adopting a 1DoF linkage that enables the spine structure to actuate quickly and precisely. To improve the speed provided by the proposed spine structure further, there are a few promising methods for increasing the force generated at the foot by actuating the spine structure in addition to enlarging the foot range of motion further. In this study, we defined the parameters of linkage in the proposed spine structure to imitate feline pelvic motion. In the future, we plan to explore parameters that maximize both the force generated at the foot and foot range of motion.

## Data Availability

The original contributions presented in the study are included in the article/Supplementary Material, further inquiries can be directed to the corresponding author.

## Footnotes

1
https://www.bostondynamics.com/spot

2
https://www.anybotics.com/anymal-autonomous-legged-robot
