# Supplementary material for: High-speed running quadruped robot with a multi-joint spine adopting a 1DoF closed-loop linkage
Source: Front Robot AI. 2023 Mar 24;10:1148816. doi: 10.3389/frobt.2023.1148816 (PMC10084478; doi:10.3389/frobt.2023.1148816)
Supplement: Supplementary file 1 [file DataSheet1.PDF]

Supplementary Material

1 PELVIC MOTION IN FELINE ANIMALS

Figure S1 represents the parts of the spine where actuation mainly occurs in feline animals. The lumbar vertebrae consist of seven segments and connect to the pelvis via the sacrum. The each "Ln" mean n-th lumbar vertebra. We defined the length of the pelvis as the distance between the "L7-P" joint and the hip joint, projected in the sagittal plane. Based on the study of Macpherson and Ye (Macpherson and Ye, 1998), we estimated the length of each Ln and the length of the pelvis of a domestic cat with a body length of approximately 350 mm. Table S1 represents each length. The angles formed by each segment were estimated based on the previous study (Macpherson and Ye, 1998; English, 1980). Table S2 represents each angle. The maximum and minimum angles mean the posture when the spine fully extends and fully flexes, respectively. From the Macpherson and Ye study, we defined "stance at preferred distance" posture as spine extension, and the posture when each angle had its maximum value on the ventroflexion side as spine flexion. We referred to most of the angle values from the Macpherson and Ye study, but some values were modified from the English study.

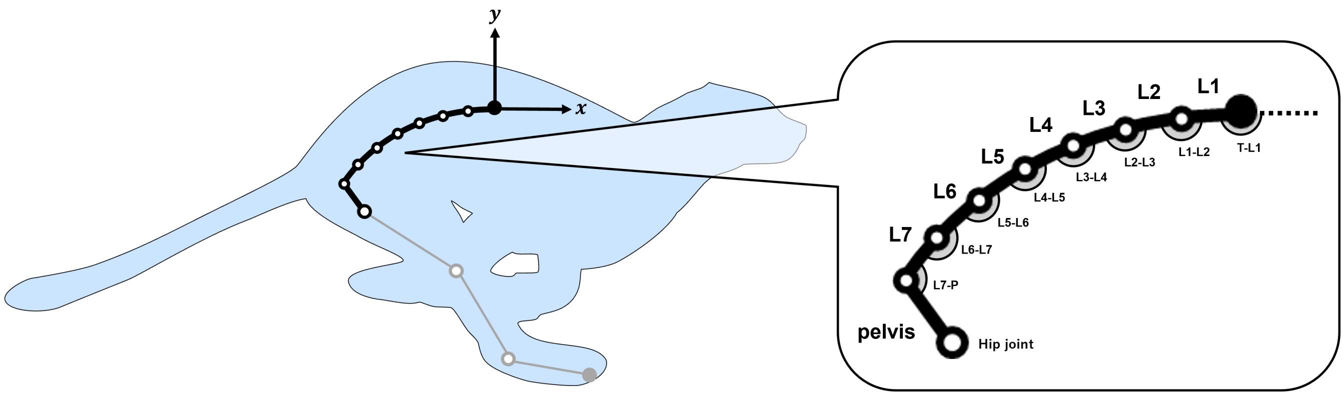

Figure S1. The parameters in the feline spine.

Table S1. Length of each segment

Property	Length [mm]
L1	15.8
L2	19.9
L3	18.3
L4	21.1
L5	23.4
L6	21.9
L7	19.8
Pelvis	34.4

Table S2. Angle formed by each segment

Property	Maximum angle [deg]	Minimum angle [deg]
T-L1	180.0	174.3
L1-L2	175.4	169.2
L2-L3	178.4	168.7
L3-L4	180.2	171.5
L4-L5	183.5	170.0
L5-L6	183.7	170.0
L6-L7	181.7	170.0
L7-P	135.8	132.9

2 THE SHAPE OF THE FOUR-BAR LINKAGE

When the coordinates of each vertex of the four-bar linkage compute, we get two solution combinations of coordinates for each vertex. In this section, we prove that one solution combination corresponds to a cross-four-bar linkage and the other corresponds to an open-four-bar linkage. Figure S2 presents the cross-four-bar linkage OABC and the open-four-bar linkage $\mathrm{OAB'C}$. We set $\mathrm{OA} = g$, $\mathrm{AB} = \mathrm{AB'} = w$, $\mathrm{BC} = \mathrm{B'C} = z$, and $\mathrm{CO} = u$. Each θ is an angle corresponding to the x-negative axis that takes positive values clockwise and negative values counterclockwise with respect to this axis.

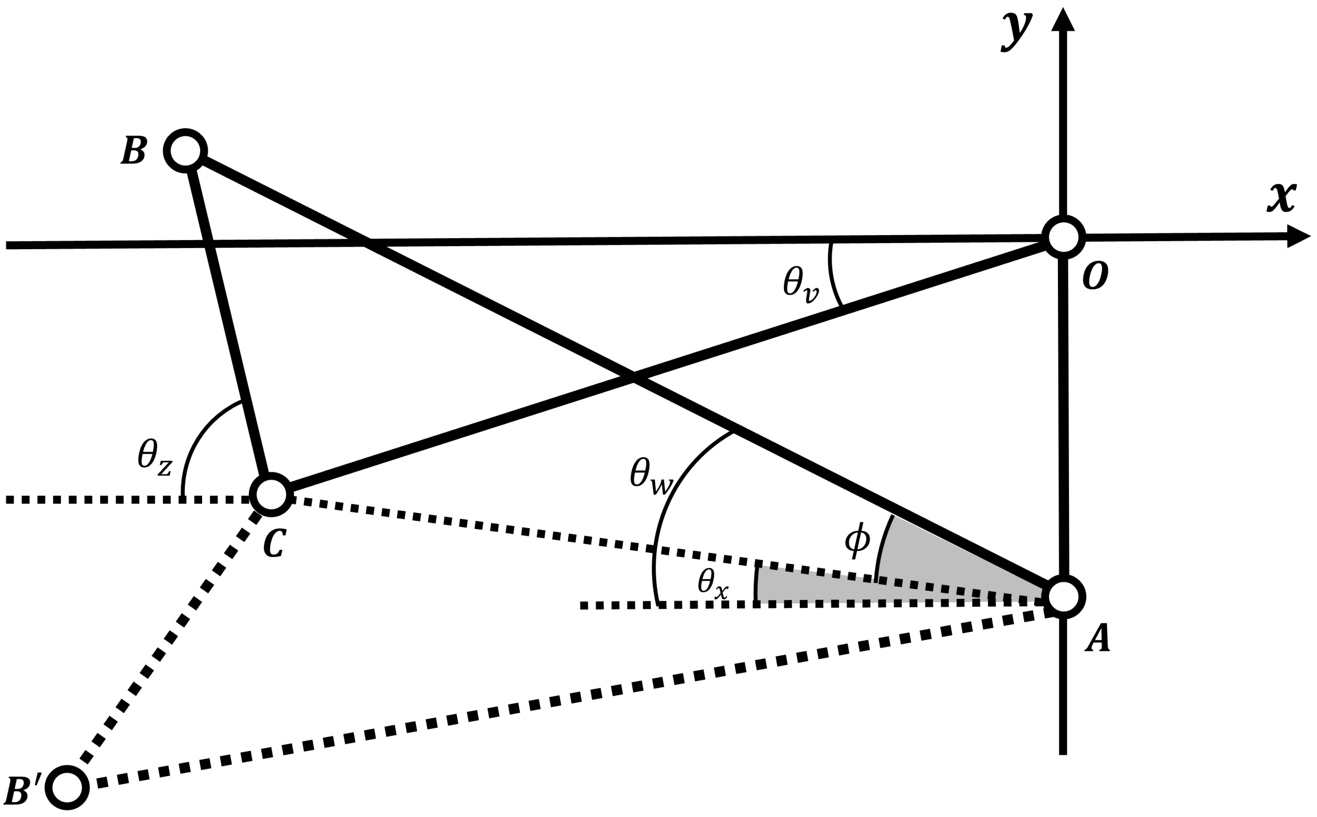

Figure S2. The cross-four-bar linkage OABC and the open-four-bar linkage $\mathrm{OAB'C}$.

First, based on the angle θ_v, we calculated the angle θ_w and the angle θ_z in the cross-four-bar linkage OABC. Equations based on the constraints of the four-bar linkage OABC can be defined as follows:

$$u\cos(\pi+\theta_v)+z\cos(\pi+\theta_z)=w\cos(\pi+\theta_w), \tag{S1}$$

$$u\sin(\pi+\theta_v)+z\sin(\pi+\theta_z)=w\sin(\pi+\theta_w)-g. \tag{S2}$$

Eqs. (S1) and (S2) can be rewritten based on θ_z as follows:

$$z\cos\theta_z=w\cos\theta_w-u\cos\theta_v, \tag{S3}$$

$$z\sin\theta_z=w\sin\theta_w-u\sin\theta_v+g. \tag{S4}$$

By combining equations Eqs. (S3) and (S4) to remove θ_z, the constraint of the four-bar linkage OABC can be described as follows:

$$z^2=g^2+u^2+w^2+(2gw-2uw\sin\theta_v)\sin\theta_w-2uw\cos\theta_v\cos\theta_w-2gu\sin\theta_v. \tag{S5}$$

By setting $t=\tan\frac{\theta_w}{2}$, $\cos\theta_w$ and $\sin\theta_w$ are expressed as follows:

$$\cos\theta_w=\frac{1-t^2}{1+t^2}, \tag{S6}$$

$$\sin\theta_w=\frac{2t}{1+t^2}. \tag{S7}$$

By using Eqs. (S6) and (S7), Eq. (S5) can be rewritten as follows:

$$(g^2+u^2+w^2-z^2-2gu\sin\theta_v+2uw\cos\theta_v)t^2+4w(g-u\sin\theta_v)t+g^2+u^2+w^2-z^2-2gu\sin\theta_v-2uw\cos\theta_v=0. \tag{S8}$$

To simplify Eq. S8, we define α, β, γ as follows:

$$\alpha=g^2+u^2+w^2-z^2-2gu\sin\theta_v+2uw\cos\theta_v, \tag{S9}$$

$$\beta=2w(g-u\sin\theta_v), \tag{S10}$$

$$\gamma=g^2+u^2+w^2-z^2-2gu\sin\theta_v-2uw\cos\theta_v. \tag{S11}$$

Based on Eqs. (S9), (S10), and (S11), Eq. (S8) can be rewritten as follows:

$$t=\frac{-\beta\pm\sqrt{\beta^2-\alpha\gamma}}{\alpha}. \tag{S12}$$

θ_w can be calculated as follows:

$$\theta_w=2\tan^{-1}\frac{-\beta\pm\sqrt{\beta^2-\alpha\gamma}}{\alpha}. \tag{S13}$$

By combining Eqs. (S3), (S4), and (S13), θ_z can be calculated as follows:

$$\theta_z=\tan^{-1}\frac{w\sin\theta_w-u\sin\theta_v+g}{w\cos\theta_w-u\cos\theta_v}. \tag{S14}$$

Based on Eq. (S13), θ_w has two solutions. One solution corresponds to the cross-four-bar linkage OABC and the other to the open-four-bar linkage $\mathrm{OAB'C}$.

Next, to find the correspondence between the solution and the shape of the four-bar linkage, we add a line AC and make triangles ABC and $\mathrm{AB'C}$. Because the lengths of each link are $\mathrm{AB} = \mathrm{AB'}$ and $\mathrm{BC} = \mathrm{B'C}$, the angles formed between link AB and AC, and link $\mathrm{AB'}$ and AC have the same value, ϕ. The angle ϕ is always positive value. In triangle ABC and triangle OAC, the constraints based on the cosine theorem are described as follows:

$$z^2 = x^2 + w^2 - 2xw\cos\phi, \tag{S15}$$

$$x^2 = g^2 + u^2 - 2gu\cos(\frac{\pi}{2} - \theta_v). \tag{S16}$$

The constraints base on the line AC can be written as follows:

$$u\cos(\pi + \theta_v) = x\cos\theta_x, \tag{S17}$$

$$u\sin(\pi + \theta_v) = x\sin\theta_x - g. \tag{S18}$$

By combining Eqs. (S15), (S16), (S17), and (S18), Eqs. (S9), (S10), and (S11) can be rewritten as follows:

$$\alpha = 2xw(\cos\theta_x + \cos\phi), \tag{S19}$$

$$\beta = -2xw\sin\theta_x, \tag{S20}$$

$$\gamma = -2xw(\cos\theta_x - \cos\phi). \tag{S21}$$

By substituting Eqs. (S19), (S20), and (S21) in Eq. (S12), we get the equation as follows:

$$t = \frac{\sin\theta_x \pm \sin\phi}{\cos\theta_x + \cos\phi}. \tag{S22}$$

To simplify Eq. (S22), we set $\cos\theta_x$, $\sin\theta_x$, $\cos\theta_\phi$, and $\sin\theta_\phi$ as follows:

$$\cos\theta_x = \frac{1 - \tan\frac{\theta_x}{2}^2}{1 + \tan\frac{\theta_x}{2}^2}, \tag{S23}$$

$$\sin\theta_x = \frac{2\tan\frac{\theta_x}{2}}{1 + \tan\frac{\theta_x}{2}^2}, \tag{S24}$$

$$\cos\theta_\phi = \frac{1 - \tan\frac{\theta_\phi}{2}^2}{1 + \tan\frac{\theta_\phi}{2}^2}, \tag{S25}$$

$$\sin\theta_\phi = \frac{2\tan\frac{\theta_\phi}{2}}{1 + \tan\frac{\theta_\phi}{2}^2}. \tag{S26}$$

By combining Eqs. (S23), (S24), (S25), and (S26), Eq. (S22) can be rewritten as follows:

$$t = \tan(\frac{\theta_x}{2} \pm \frac{\theta_\phi}{2}). \tag{S27}$$

Because we set $t = \tan\frac{\theta_w}{2}$, θ_w can be described as follows:

$$\theta_w = \theta_x \pm \phi. \tag{S28}$$

Considering that the angle ϕ takes a positive value, the part corresponding to the " $\pm$" in Eq. (S28) is " $+$" when the shape of the four-bar linkage OABC opens and " $-$" when it crosses. θ_w when selecting the cross-four-bar linkage OABC can be determined as follows:

$$\theta_w = 2\tan^{-1}\frac{-\beta - \sqrt{\beta^2 - \alpha\gamma}}{\alpha}. \tag{S29}$$

θ_w when selecting the open-four-bar linkage $\mathrm{OAB'C}$ can be determined as follows:

$$\theta_w = 2\tan^{-1}\frac{-\beta + \sqrt{\beta^2 - \alpha\gamma}}{\alpha}. \tag{S30}$$

This proof of the correspondence between the solution and the shape of the four-bar linkage is depending on the arrangement of the parameters shown in Figure S2. Note that the correspondence shown above does not necessarily match if the arrangement of the parameters is changed from that in Figure S2.

3 DURATION TIME OF EACH PHASE

To determine the representative running speed of the robot equipped with each spine structure, we conducted a parameter search by varying the duration of each phase and measuring the running speed. We fixed the period T at 330 ms and vary the ratios occupied by the stance, liftoff, swing, and touchdown phases. We defined the percentage of each phase as P_{Stance}, P_{Liftoff}, P_{Swing}, and $P_{\text{Touchdown}}$. We selected and combined each P from the following so that $P_{\text{Stance}} + P_{\text{Liftoff}} + P_{\text{Swing}} + P_{\text{Touchdown}} = 100$.

$$P_{\text{Stance}} \in \{35, 45\}$$
$$P_{\text{Liftoff}} \in \{5, 15, 25, 35, 45\}$$
$$P_{\text{Swing}} \in \{5, 15, 25, 35, 45\}$$
$$P_{\text{Touchdown}} \in \{5, 15, 25, 35, 45\}$$

We changed the value in increments of 10 and exclude combinations that the robot expects to run slower than the representative running speed to reduce the number of combinations. The duration of each phase is the period T multiplied by P of each phase, rounded down to the nearest whole number.

The experimental environment for measuring the running speed of the robot was the same as in the main text. Table S2 presents the results of the parameter search. The percentage and duration of each phase are listed in the order of stance, liftoff, swing, and touchdown phase. When the percentage of each phase is $P_{\text{Stance}} = 35$, $P_{\text{Liftoff}} = 25$, $P_{\text{Swing}} = 35$, $P_{\text{Touchdown}} = 5$, the robot running speed is one of the fastest for each spine structure. Based on this result, we determined $T_{\text{Stance}} = 115$ ms, $T_{\text{Liftoff}} = 82$ ms, $T_{\text{Swing}} = 115$ ms, and $T_{\text{Touchdown}} = 16$ ms to show the representative running speed of the robot equipped with each spine structure.

Table S3. Result of the parameter search

Percentage [%]	Duration time [ms]	Proposed spine structure [km/h]	Single-joint spine structure [km/h]
45-45-05-05	148-148-016-016	-*	-*
45-35-15-05	148-115-049-016	1.8	1.0
45-35-05-15	148-115-016-049	2.0	1.2
45-25-25-05	148-082-082-016	3.3	1.8
45-25-15-15	148-082-049-049	4.2	2.2
45-25-05-25	148-082-016-082	-*	3.0
45-15-35-05	148-049-115-016	2.3	2.1
45-15-25-15	148-049-082-049	2.7	2.4
45-15-15-25	148-049-049-082	1.8	2.3
45-15-05-35	148-049-016-115	1.8	2.4
45-05-45-05	148-016-148-016	1.0	1.7
45-05-35-15	148-016-115-049	-*	1.8
45-05-25-25	148-016-082-082	1.0	1.9
45-05-15-35	148-016-049-115	1.0	1.5
45-05-05-45	148-016-016-148	1.0	1.4
35-45-15-05	115-148-049-016	-*	1.1
35-45-05-15	115-148-016-049	1.7	1.2
35-35-25-05	115-115-082-016	4.1	2.4
35-35-15-15	115-115-049-049	2.4	2.4
35-35-05-25	115-115-016-082	-*	3.0
35-25-35-05	115-082-115-016	5.8	3.0
35-25-25-15	115-082-082-049	4.8	2.8
35-25-15-25	115-082-049-082	4.7	2.8
35-25-05-35	115-082-016-115	4.7	3.0
35-15-45-05	115-049-148-016	2.4	2.8
35-15-35-15	115-049-115-049	-*	2.0
35-15-25-25	115-049-082-082	1.0	2.2
35-15-15-35	115-049-049-115	1.0	2.2
35-15-05-45	115-049-016-148	1.0	2.1
35-05-45-15	115-016-148-049	-*	1.9
35-05-35-25	115-016-115-082	-*	1.7
35-05-25-35	115-016-082-115	-*	1.8
35-05-15-45	115-016-049-148	-*	-*

* The robot moved at less than the minimum speed that could be set (1.0 km/h) on the treadmill, or the running speed was too unstable to measure 10 s run on the treadmill.

REFERENCES

English, A. W. (1980). The functions of the lumbar spine during stepping in the cat. *Journal of Morphology* 165

Macpherson, J. and Ye, Y. (1998). The cat vertebral column: stance configuration and range of motion. *Experimental brain research* 119, 324–332
